# Integrated time-series biochemical, transcriptomic, and metabolomic analyses reveal key metabolites and signaling pathways in the liver of the Chinese soft-shelled turtle (*Pelodiscus sinensis*) against *Aeromonas hydrophila* infection

**DOI:** 10.3389/fimmu.2024.1376860

**Published:** 2024-05-10

**Authors:** Liqin Ji, Chen Chen, Junxian Zhu, Xiaoyou Hong, Xiaoli Liu, Chengqing Wei, Xinping Zhu, Wei Li

**Affiliations:** Key Laboratory of Tropical and Subtropical Fishery Resources Application and Cultivation, Ministry of Agriculture and Rural Affairs, Pearl River Fisheries Research Institute, Chinese Academy of Fishery Sciences, Guangzhou, Guangdong, China

**Keywords:** Chinese soft-shelled turtle, plasma parameters, transcriptome, metabolome, liver, *Aeromonas hydrophila*

## Abstract

**Introduction:**

*Aeromonas hydrophila*, a bacterium widely distributed in the natural environment, causes multiple diseases in various animals. Exploring the mechanism of the host defense against *A. hydrophila* can help develop efficient strategies against *Aeromonas* infection.

**Methods:**

Herein, we investigated the temporal influence of A. hydrophila on the Chinese soft-shelled turtle, an economically important species, at the biochemical, transcriptomic, and metabolomic levels. Plasma parameters were detected with the test kits. Transcriptome and metabolome were respectively applied to screen the differentially expressed genes and metabolites.

**Results:**

The contents or activities of these plasma parameters were significantly increased at 24 hpi and declined at 96 hpi, indicating that 24 and 96 hpi were two important time points during infection. Totals of 3121 and 274 differentially expressed genes (DEGs) from the transcriptome while 74 and 91 differentially abundant metabolites (DAMs) from the metabolome were detected at 24 and 96 hpi. The top DEGs at 24 hpi included *Ccl2, Ccl3, Ccl4, Il1β, Il6, Il7, Il15, Tnf,* and *Tnfr1* while *Zap70, Cd3g, Cd8a, Itk, Pik3r3, Cd247, Malt1,* and *Cd4* were the most abundant at 96 hpi. The predominant DAMs included O-phospho-L-serine, γ-Aminobutyric acid, orotate, L-tyrosine, and L-tryptophan at 24 hpi, as well as L-glutamic acid, L-arginine, glutathione, glutathione disulfide, and citric acid at 96 hpi.

**Discussion:**

The combined analysis of DEGs and DAMs revealed that tryptophan metabolism, nicotinate and nicotinamide metabolism, as well as starch and sucrose metabolism, were the most important signaling pathways at the early infective stage while tyrosine metabolism, pyrimidine metabolism, as well as alanine, aspartate and glutamate metabolism were the most crucial pathways at the later stage. In general, our results indicated that the Chinese soft-shelled turtle displays stage-specific physiological responses to resist *A. hydrophila* infection.

## 1 Introduction

*Aeromonas* species are widely distributed in the soil and in aquatic habitats such as sediment, feces, and drinking water (1, 2). *Aeromonas hydrophila* is a freshwater, facultatively anaerobic, Gram-negative bacterium that can infect various species, including shrimp, fishes, amphibians, reptiles, and mammals (3, 4). *Aeromonas hydrophila* survives in the temperature range of 0°C–45°C, with the optimal range of 22°C–32°C (5). Multiple stress conditions such as temperature fluctuation, water pollution, overcrowding, low dissolved oxygen, and concurrence with other pathogens may increase the vulnerability to *A. hydrophila* infection, indicating that *A. hydrophila* is an opportunistic pathogen (6, 7).

Turtles are common carriers of pathogenic *A. hydrophila*, and humans are at risk of infection by physical contact (8). The turtles have a unique evolutionary status as secondary aquatic reptiles; therefore, their response mechanism against pathogens may be distinct from those of mammals and fishes (9, 10). The Chinese soft-shelled turtle (*Pelodiscus sinensis*) is an economically important reptile in East Asian countries, being especially popular in China and Japan, owing to its food and medicinal value for humans. The consumption of turtles is deemed beneficial for enhancing immunity, anti-aging, and relieving cardiocerebrovascular diseases based on ancient Chinese medicine theory (11). *Aeromonas hydrophila* infection has led to more than 15 diseases in turtles, such as keratitis, red-neck disease, septicemia, and furunculosis (12, 13). These diseases account for approximately 60% of the total disease cases in turtles and have led to severe economic losses (14). Therefore, research on the response mechanisms of Chinese soft-shelled turtles against *A. hydrophila* is needed for the prevention of related diseases.

Previous studies on the immune response of Chinese soft-shelled turtles to *A. hydrophila* emphasized the spleen, a typical immune organ (10, 14). The immune response of the liver has received comparatively less attention in aquatic animals. Traditionally, the liver plays crucial roles in lipid metabolism, detoxification, and glycogen storage (15). During the past several decades, the liver has gradually been perceived as an organ with critical functions in immunity. MacParland et al. have performed single-cell RNA sequencing of human liver samples, providing a map of the human hepatic immune microenvironment (16). These immune cells include innate forms such as Kupffer, dendritic, and natural killer (NK) cells as well as adaptive immune cells such as CD4^+^, CD8^+^ T cells, and B cells (16). In addition, the gut–liver axis, involving the intestinal microbiome and the hepatic immune system, plays important roles in the immune response of mammals and fishes (17). For example, Wu et al. produced a sophisticated profile of fish gut–liver immunity during both homeostasis and inflammation in healthy and infected tilapia (18). Therefore, comprehensively exploring the immune mechanism of the liver in resisting bacterial infection would extend our knowledge of immune processes in Chinese soft-shelled turtles.

Hematology can be used to monitor the physiological status of the liver (19). The serum biochemical parameters often provide the first clue of the presence of liver pathology. For example, glutamate pyruvate transaminase (GPT) and glutamic oxalacetic transaminase (GOT), as ubiquitous transaminases, are two clinical biomarkers of hepatic health. These two enzymes, generally existing in hepatocytes, are secreted into the blood when the permeability of the hepatic cell membrane becomes damaged (20). Alkaline phosphatase (AKP), existing in macrophage lysosomes, has important functions in immunity, as it can mitigate the negative effects of lipopolysaccharides and alleviate inflammation (21). One of the functions of catalase (CAT), as an endogenous antioxidant enzyme, is to remove excessive reactive oxygen species (ROS) generated by bacterial infection (22). Malonaldehyde (MDA) is the final product of lipid peroxidation, and its concentration is associated with the toxic effects of ROS (23). Thus, the analysis of plasma parameters can help to assess the health status of individuals.

Metabolites are involved in regulating the immune response or signal pathways of the hosts challenged by pathogens or external stresses (24–26). For example, the liver metabolome of yellow catfish showed that iron metabolism modulated by hepcidin could contribute to the defense against *Aeromonas veronii* infection (24). The metabolomic analysis of the gill in *Oreochromis mossambicus* found that the levels of amino acids, osmolytes, and energy substances are significantly affected by osmotic stresses (25). The spleen metabolome of *Paralichthys olivaceus* identified numerous metabolites responding to *Edwardsiella tarda* invasion and temperature alteration, including L-methionine and UDP-glucose (26). Recently, it has been demonstrated that a combination of metabolome and transcriptome can provide more comprehensive information than a single omics technique (27). For example, a combined analysis of gut transcriptome and metabolome in zebrafish was employed to explore the potential anti-inflammatory mechanisms of gallic acid in alleviating soybean meal-induced enteritis (28). The integration of two omics identified the key pathways in the liver of the yellow catfish responding to *A. veronii* infection related to ascites, body surface ulcers, and hemorrhagic septicemia (29). A two-omics analysis of the liver in *Acipenser dabryanus* revealed the molecular mechanisms for dealing with thermal stress (30).

Our study detected plasma biochemical indicators, the hepatic transcriptome, and the metabolome of Chinese soft-shelled turtles challenged by *A. hydrophila* at different infective stages. These findings deciphered the immunological and metabolic mechanisms of hepatic tissue responding to bacterial infection in Chinese soft-shelled turtles. The screened metabolites and signaling pathways responding to *A. hydrophila* provided valuable strategies for preventing bacterial disease in Chinese soft-shelled turtles.

## 2 Materials and methods

### 2.1 Experimental animals and bacterial infection

A total of 80 healthy Chinese soft-shelled turtles with an average body weight of 452 ± 47 g were purchased from Huizhou Wealth Xing Industrial Co., Ltd. (Huizhou, China). The experiments were performed in the Guangzhou Aquatic Thoroughbred Base of the Pearl River Fisheries Research Institute (Guangzhou, China). These animals were acclimated for 2 weeks in 16 acrylic tanks (1 m × 1 m × 0.25 m) and fed commercial pellet diets with product number 0081 (Guangdong Nutriera Group Co., Ltd., Guangzhou, China) twice a day at 9:00 and 16:00. Turtles were fed by hand to apparent satiation (over 90% of the individuals had no apparent feeding behavior, and over 5 g of pellets reached the bottom of the tank). The residual pellets were siphoned out 1 h after each meal. During the trial, the water temperature was kept at 27°C ± 1°C via air conditioning. The pH, dissolved oxygen, NH_3_-N, NO_2_^–^, and alkalinity of the water were maintained at 8.0 ± 0.4, 6.0 ± 1.7 mg/L, 4.0 ± 1.2 mg/L, 1.0 ± 0.4 mg/L, and 45 ± 4, respectively. Approximately one-third of the tank’s water was renewed once a week during acclimation. After the acclimation, the turtles were fasted for 48 h prior to the bacterial challenge test. Healthy individuals that had no clinical signs of disease on the surface were chosen for the bacterial infection.

*Aeromonas hydrophila* originally isolated from a diseased Chinese soft-shell turtle was kindly gifted by Dr. Aiping Tan from the Pearl River Fisheries Research Institute, Chinese Academy of Fishery Sciences (Guangzhou, China). *Aeromonas hydrophila* was identified by 16S rDNA sequence analysis before the challenge test. The bacteria were cultured in brain heart infusion (BHI) broth at 28°C for 24 h and then centrifuged at 2,500×*g* for 10 min, and the supernatant was discarded. The bacteria were resuspended in sterile 0.85% NaCl (31), adjusting the concentration to 8.0 × 10^8^ CFU/mL in accordance with the 0.5 McFarland standard. A concentration of 8.0 × 10^8^ CFU/mL was the median lethal concentration for turtles during *A. hydrophila* infection in a preliminary experiment.

The turbidity was adjusted using a turbidimeter (BioMerieux, USA). Sixty turtles intraperitoneally injected with 500 μL of freshly prepared *A. hydrophila* (8.0 × 10^8^ CFU/mL) comprised the infected group (IG); meanwhile, 10 turtles injected with 500 μL of 0.85% NaCl were regarded as the control group (CG).

### 2.2 Sample collection

Sampling (*n* = 6) took place at 0, 12, 24, 48, and 96 h post-infection (hpi). Individuals from the control group injected with an equal volume of 0.85% NaCl were considered as the 0-hpi sample. The turtles were rapidly anesthetized by immersion in 250 mg/L of MS222 solution prior to dissection. The blood samples for biochemical analysis were collected from the jugular vein using a 5-mL vacutainer with heparin sodium and kept at 4°C for 5 h. Then, the blood samples were centrifuged at 4,000×*g* for 20 min at 4°C to obtain plasma, which was stored at −80°C for analysis of plasma biochemical parameters. The collected liver tissues were snap-frozen in liquid nitrogen and stored at −80°C for subsequent detection.

### 2.3 Plasma biochemical parameters

The concentrations or activities of plasma malonaldehyde (MDA), glucose (GLU), catalase (CAT), glutamate pyruvate transaminase (GPT), glutamic oxalacetic transaminase (GOT), and alkaline phosphatase (AKP) were assayed using test kits (Nanjing Jiancheng Bioengineering Institute, Nanjing, China), with six biological repeats at each time point (*n* = 6) (30).

### 2.4 RNA extraction, library preparation, and RNA sequencing

Liver tissues from three time points (0, 24, and 96 hpi) with five biological repeats at each time point (*n* = 5) were analyzed for RNA sequencing. Total RNA was extracted from the liver tissues using TRIzol reagent (Ambion Life Technologies, Carlsbad, USA) and treated with RNase-free DNase I (Qiagen, Germantown, USA) at 37°C for 1 h to remove residual genomic DNA. RNA integrity and quantity were assessed by electrophoresis in 1% agarose gels and a NanoDrop 2000 spectrometer (Thermo Fisher Scientific, Wilmington, USA) with A260/A280 ratios between 1.8 and 2.0, respectively.

The RNA-seq libraries were constructed using 5 mg of RNA and were paired-end sequenced on an Illumina HiSeq 4000 sequencing platform (Illumina, San Diego, USA) by Gene Denovo Biotechnology Co. Ltd. (Guangzhou, China). The clean data were screened from the raw reads by removing adapter sequences, poly-N sequences, and low-quality sequences with fastp 0.18.0 software (32). Effective reads were mapped to the *P. sinensis* genome (https://www.ncbi.nlm.nih.gov/datasets/genome/GCF_000230535.1/) using HISAT2 (33). The sequencing datasets are available from the Short Read Archive (SRA) of NCBI with accession number SUB14127776.

The expression levels of the genes in different cDNA libraries were calculated with the fragments per kilobase of exon per million mapped fragments (FPKM) values (34). Differentially expressed genes (DEGs) were identified by DESeq2 (35) and defined by fold change >2 and false discovery rate (FDR) <0.05. The DEGs were further subjected to GO analysis (36) and KEGG enrichment analysis (37).

### 2.5 Metabolomic analysis

#### 2.5.1 Metabolic extraction

Liver tissues (50 mg) of five individuals (*n* = 5) from each of the three time points (0, 24, and 96 hpi) were collected for metabolomic analysis. Liver tissues were homogenized with 500 μL of prechilled 70% methanol containing internal standard (L-2-chlorophenylalanine, 1 ppm) at 30 Hz for 30 min in an ice bath. Then, the mixture was vortexed for 5 min and incubated on ice for 15 min. The mixture was centrifuged at 14,000 rpm at 4°C for 20 min. The supernatant was dried in a vacuum centrifuge and redissolved in 100 µL of acetonitrile solvent (acetonitrile/water = 1:1) for LC–MS/MS analysis. Finally, 60 µL of the supernatant was analyzed by a liquid chromatography tandem-mass spectrometry system (LC–MS) (38).

#### 2.5.2 LC–MS analysis

High-performance liquid chromatography (HPLC) separation was performed with a 1290 Infinity LC UHPLC System (Agilent Technologies, Waldbronn, Germany). To determine stability and reliability, quality control (QC) samples were prepared by pooling an equal aliquot of each sample and analyzing these together with the experimental samples. Mass spectrometry was carried out by a Q Exactive™ HF mass spectrometer (Thermo Scientific, San Jose, USA) after the separation of the samples by HPLC. Each sample was operated in positive/negative polarity mode by electrospray ionization (ESI) with the following parameters: spray voltage, 3.5 kV; source temperature, 320°C; sheath gas flow rate, 45 arb; and aux gas flow rate, 15 arb.

Peak alignment, peak picking, and metabolic quantitation were performed using the Compound Discoverer 3.1 program. Publicly available metabolite databases, including KNApSAcK (http://kanaya.naist.jp/KNApSAcK/), MassBank (http://www.massbank.jp/), HMDB (http://www.hmdb.ca/), and METLIN (http://metlin.scripps.edu/index.php), were utilized for annotating the metabolites. Multivariate statistical analyses were carried out using the MetaboAnalystR (V1.0.1) R package, including principal component analysis (PCA), partial least squares discriminant analysis (PLS-DA), and orthogonal partial least squares discriminant analysis (OPLS-DA). The criteria for differential abundance metabolites (DAMs) were variable importance in projection (VIP) >1 and *P <*0.05 in an independent sample *t*-test. These were selected as differential metabolites for pairwise comparisons for three groups, namely, comparisons of CG vs. IG24, CG vs. IG24, and IG24 vs. IG96.

### 2.6 Correlation analysis of metabolomic and transcriptomic data

DEGs (FDR < 0.05 and |log2FC| > 1) and DAMs (VIP > 1 and *P* < 0.05) were integrated for the pairwise comparisons listed above. A pathway model, a Pearson model, and an O2PLS model were established to analyze the association between the transcriptome and metabolome. All DEGs and DAMs were mapped to the KEGG pathway database to obtain their associations with metabolic pathways. Pearson correlation coefficient (PCC) and the relevant *P*-values of the Pearson model were used to evaluate the correlation between the DEGs and DAMs. Those with |PCC| >0.80 and *P <*0.05 were considered to be significant.

O2PLS models were calculated using the OmicsPLS package for R. This method decomposed the variation present in the two data matrices into three parts: the joint variation between the two datasets, the orthogonal variation unique to each dataset, and noise (39). In this model, joint loading plots of the transcriptome and metabolome were constructed to represent the correlation between metabolites and genes. In the loading plots, a higher absolute value represented a better correlation between genes and metabolites, meaning that the genes (metabolites) far from the origin were more highly associated with metabolites (genes) (39).

### 2.7 Validation of transcriptomic data

Total RNA from the 0-, 24-, and 96-hpi groups was used for quantitative real-time PCR (qRT-PCR) validation. The RNA for qRT-PCR validation was the same as that employed in constructing the RNA-seq library.

Before the formal qRT-PCR experiment, we utilized a no-RT negative control (cDNA reverse-transcribed from the reactions without reverse transcriptase) for each cDNA sample to exclude the interference of the genomic DNA in the qRT-PCR. The qRT-PCR was carried out in the ABI StepOnePlus System (Applied Biosystems, Foster, USA) using iTaq™ Universal SYBR^®^ Green Supermix (TaKaRa, Dalian, China). The 20-μL reaction mix in each well contained 2 μL of diluted cDNA (~1 μg), 10 μL of 2× SYBR Green Master Mix, 4 μM of each primer, 0.4 μL of ROX reference dye, and RNase-free water to a final volume of 20 μL. All reactions were run using the following program: 95°C for 5 min, followed by 40 cycles of 95°C for 5 s and 60°C for 30 s. Dissociation curve analysis was performed with the temperature gradually increasing from 63°C to 95°C to ensure the specificity of the target genes. A total of 15 DEGs were randomly selected to verify the sequencing results. The specific qRT-PCR primers designed by Primer Premier 5 software are shown in **Supplementary Table 1**. All samples were run in duplicate. The relative expression levels of the target genes were normalized with the housekeeping gene *elof1* and were calculated according to the Pfaffl method (40). Finally, the qRT-PCR results were shown as fold changes relative to the expression level of genes in the 0-hpi group.

### 2.8 Statistical analysis

All data from plasma biochemical parameter analyses were represented as the mean ± standard error (SE). Group comparisons were performed by one-way analysis of variance (ANOVA) followed by Duncan’s *post-hoc* test. A value of *P <*0.05 was considered statistically significant. To compare fold changes between qRT-PCR and DEG results, the Pearson *R* ^2^ values were calculated and plotted with GraphPad Prism 9.0 software.

### 2.9 Ethics statement

All infection and sample procedures in the experiments were performed following the Guidelines for the Care and Use of Laboratory Animals in China and were approved by the Ethics Committee of the Pearl River Fisheries Research Institute, Chinese Academy of Fishery Sciences (LAEC-PRFRI-2023-03-15).

## 3 Results

### 3.1 Changes in plasma biochemical parameters during infection

**Figure 1:**
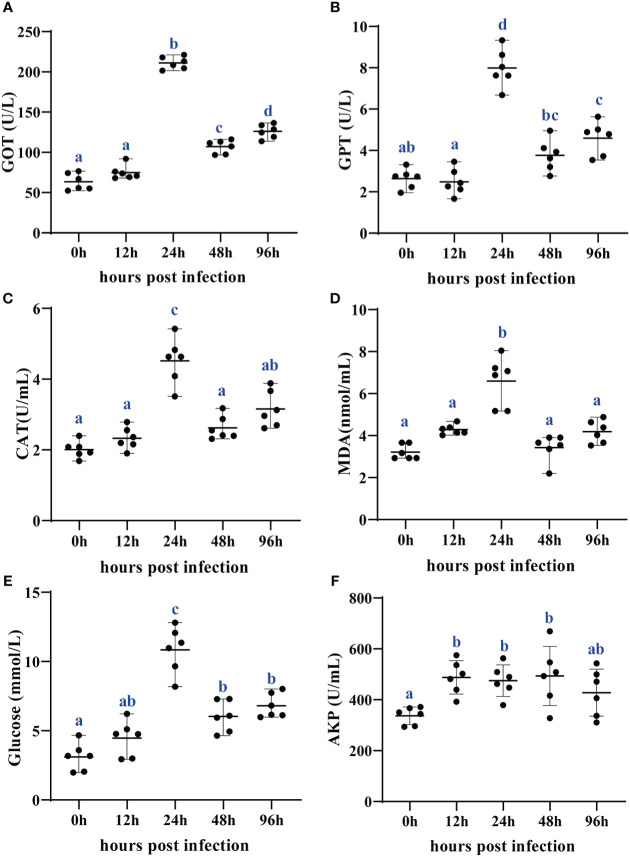
Changes of plasma GOT **(A)**, GPT **(B)**, CAT **(C)**, MDA **(D)**, glucose **(E)**, and AKP **(F)** in Chinese soft-shelled turtles at 0, 12, 24, 48, and 96 h after *Aeromonas hydrophila* infection. All data are represented as mean ± SE (*n* = 6). Different superscript letters mean significant difference (*P* < 0.05). GOT, glutamic oxalacetic transaminase; GPT, glutamate pyruvate transaminase; CAT, catalase; MDA, malonaldehyde; AKP, alkaline phosphatase.

The stress response of Chinese soft-shelled turtles to the *A. hydrophila* challenge was investigated using six plasma indices: two aminotransferases (GOT and GPT), two oxidative stress markers (CAT and MDA), one antimicrobial enzyme (AKP), and glucose (**Figure 1**). The plasma GOT and GPT activities were significantly increased to the highest levels at 24 hpi (*P* < 0.05). Plasma GOT activity at 48 hpi and 96 hpi and GPT activity at 96 hpi were higher than those at 0 hpi (*P* < 0.05). The plasma glucose levels were elevated at 24, 48, and 96 hpi compared with those at 0 hpi (*P* < 0.05) and reached the highest levels at 24 hpi. The plasma AKP activities at 12, 24, and 48 hpi were higher than those at 0 hpi (*P* < 0.05). There was no difference in AKP activity between 0 hpi and 96 hpi (*P* > 0.05). The plasma CAT activity and MDA content reached the highest levels at 24 hpi (*P* < 0.05), while there were no significant differences at other time points (*P* > 0.05).

### 3.2 The hepatic DEGs at different time points after infection

Transcriptomic sequencing of CG, IG24, and IG96 was performed with five biological replicates in each group, generating 15 RNA-seq libraries. A total of 50,274,586, 44,530,277, and 47,898,948 clean reads were obtained for the CG, IG24, and IG96 groups, respectively. Genes were mapped to the Chinese soft-shelled turtle’s genome, and the average mapping ratios for the CG, IG24, and IG96 groups were 89.35%, 88.59%, and 89.14%, respectively. The average Q20 and Q30 values in all groups were greater than 97% and 93%, respectively, indicating that the data quality was sufficient for subsequent analysis (**Supplementary Table 2**).

A hepatic RNA-seq analysis of Chinese soft-shelled turtles at 0, 24, and 96 hpi was conducted to reveal the changes in gene expression at different stages of infection (**Supplementary Table 3**, **Supplementary Figure 1**). A heatmap showed that the biological replicates in each group exhibited high repeatability, with correlation coefficients greater than 0.9 (**Supplementary Figure 1A**). PCA represented that the three groups could be separated by the first principal component (PC1) (**Supplementary Figure 1B**). PC1 and PC2, respectively, explaining 50.8% and 19.5% of the total variation, were the dominant components in discriminating the three groups. A Venn diagram exhibited 4,236 DEGs from three pairwise comparisons (**Supplementary Figure 1C**). Sixty common DEGs were detected among the three comparisons; moreover, 172 common DEGs were detected in the comparison groups of CG vs. IG24 and CG vs. IG96. For DEGs between the two groups, there were 3,121 DEGs in the comparison groups CG vs. IG24, with 1,617 being upregulated and 1,504 being downregulated, while there were 274 DEGs in the comparison groups CG vs IG96, comprising 198 upregulated and 76 downregulated DEGs (**Supplementary Figure 1D**). Moreover, there were 3,042 DEGs in the comparison groups IG24 vs. IG96, including 1,508 upregulated and 1,534 downregulated DEGs.

**Figure 2:**
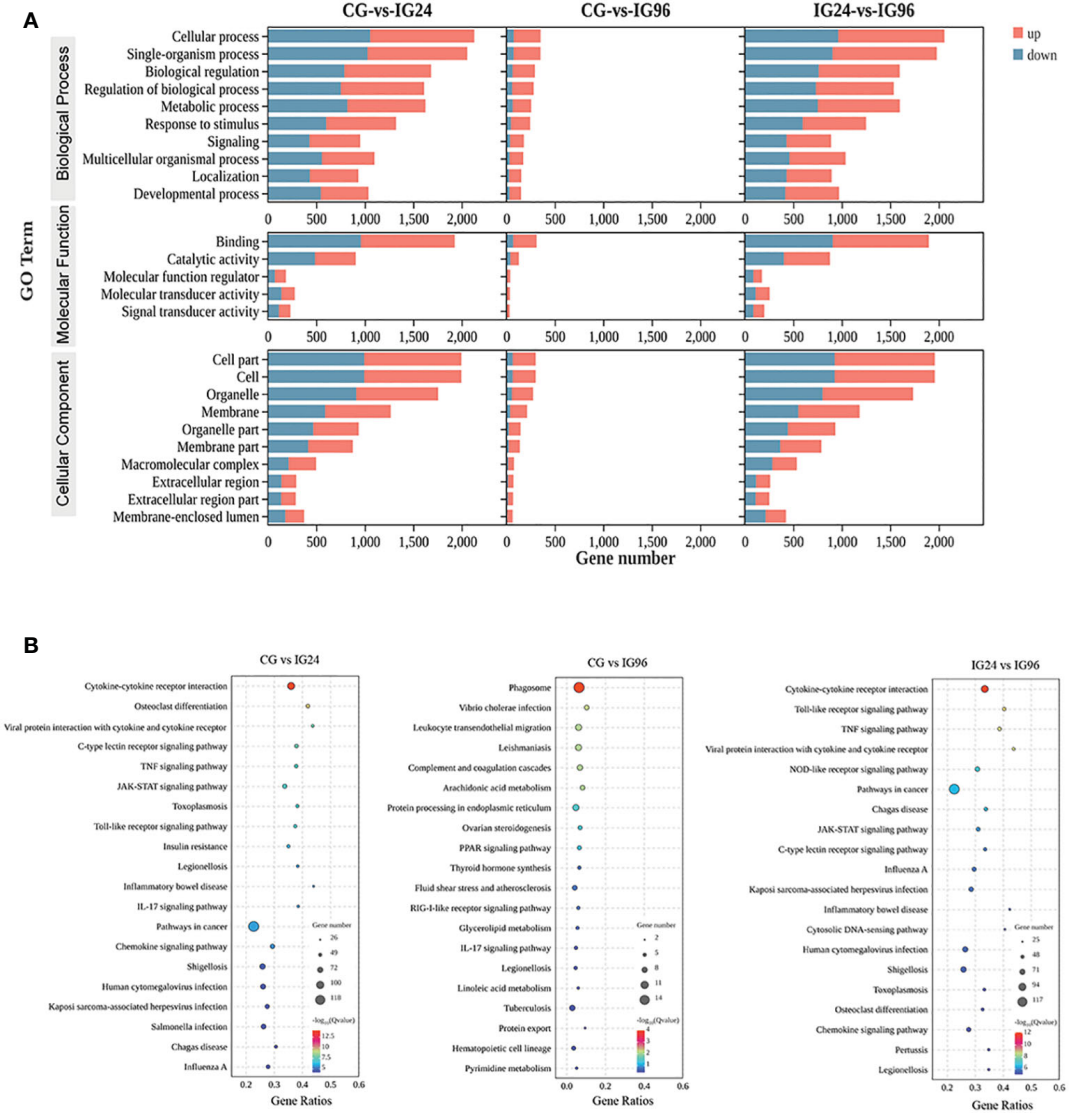
GO **(A)** and KEGG **(B)** enrichment results of differentially expressed genes (DEGs) in the liver of Chinese soft-shelled turtles at 0, 24, and 96 h after infection by *Aeromonas hydrophila*. “CG” indicates the control group, and “IG24” and “IG96” indicate the infected groups at 24 and 96 h after *A hydrophila* infection.

The functional classification of DEGs was carried out by GO and KEGG enrichment analyses (**Supplementary Table 3**, **Figure 2**). All DEGs were enriched in the three main GO categories (biological process, cellular component, and molecular function) (**Figure 2A**). For the CG vs. IG24 and IG24 vs. IG96 comparisons, the main subcategories in the biological process included cellular process (GO:0009987), single organism (GO:0044699), biological regulation (GO:0065007), and metabolic process (GO:0008152). Meanwhile, cell part (GO:0044464), cell (GO:0005623), organelle (GO:0043226), membrane (GO:0016020), and organelle part (GO:0044422) were the top 5 subcategories of cellular component. Binding (GO:0005488) and catalytic activity (GO:0003824) were the dominant subcategories in the molecular function category of the three comparisons. The KEGG pathways represented the DEG-regulated signaling pathways responding to *A. hydrophila* infection at different time points (**Figure 2B**). In the comparison groups CG vs. IG24 and IG24 vs. IG96, the DEGs were significantly enriched in cytokine-cytokine receptor interaction (ko04060), viral protein interaction with cytokine and cytokine receptor (ko04061), C-type lectin receptor signaling pathway (ko04625), tumor necrosis factor (TNF) signaling pathway (ko04668), JAK-STAT signaling pathway (ko04630), and Toll-like receptor signaling pathway (ko04620). However, the KEGG pathways in the comparison groups CG vs. IG96, different from the other pairwise comparisons, were enriched in the phagosome (ko04145), *Vibrio cholerae* infection (ko05110), leukocyte transendothelial migration (ko04670), leishmaniasis (ko05140), and the complement and coagulation cascades (ko04610).

### 3.3 Hepatic metabolic profiles at different time points after infection

**Figure 3:**
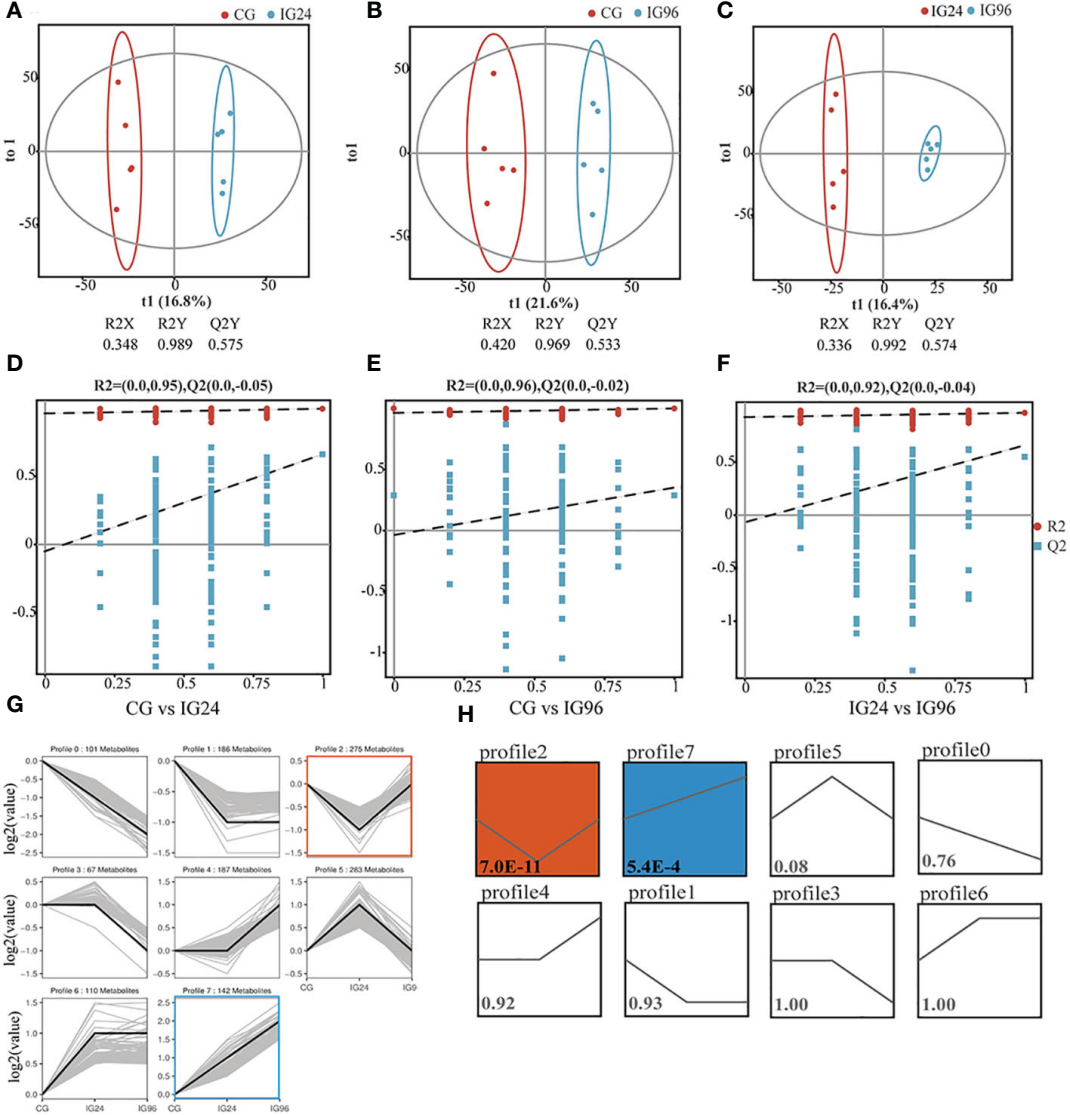
Time-course metabolomic analysis in *Aeromonas hydrophila*-infected liver. Orthogonal projection to latent structures-discriminant analysis (OPLS-DA) with corresponding cross-validation of metabolite profiles in CG vs. IG24 **(A)**, CG vs. IG96 **(B)**, and IG24 vs. IG96 **(C)**. The permutation test results of OPLS-DA in CG vs. IG24 **(D)**, CG vs. IG96 **(E)**, and IG24 vs. IG96 **(F)**. **(G)** Metabolite variation tendencies in eight cluster profiles. *X*-axis: time; *Y*-axis: log_2_(contents of metabolites in the IGs relative to CG). **(H)** The statistical significance of clustered profiles evaluated by *P*-value <0.05. The number in the square indicates the *P*-value. The two-colored profiles with *P*-value <0.05 mean the significantly clustered profiles. “CG” indicates the control group, and “IG24” and “IG96” indicate the infected groups at 24 and 96 h after *A hydrophila* infection.

To investigate the metabolic changes in the liver of Chinese soft-shelled turtles during *A. hydrophila* challenge, the widely targeted LC–MS metabolome was performed at 0, 24, and 96 hpi (**Supplementary Table 4**). Correlation analysis demonstrated the high similarity of the metabolic composition of five replicates in each group (*r* > 0.93) (**Supplementary Figure 2A**). Multivariate statistical analyses, including PCA, PLS-DA, and OPLS-DA, were applied to analyze the difference in metabolic profiles among the groups. PCA (**Supplementary Figure 2B**) and PLS-DA (**Supplementary Figures 2C-E**) indicated significant separation of the metabolic profiles among the groups. To identify the pairwise discrimination criteria between groups, an OPLS-DA model was established and verified using cross-validation and permutation tests (**Figures 3A-F**). The two parameters of cross-validation, namely, R2Y >0.96 and Q2 >0.50, indicated the goodness of fit and high predictability of the OPLS-DA model (**Figures 3A-C**). The Y-intercept of Q2 <0 from the permutation test indicated the reliability of the model (**Figures 3D-F**). Therefore, this OPLS-DA model was used to identify pairwise differences between the groups for subsequent analysis. Then, 1,408 metabolites from three time points, categorized by similar variation trends, were clustered into eight profiles (**Figures 3G, H**). Profiles 2 and 5, respectively, contained 275 and 283 metabolites, showing a significant change at 24 hpi. Profiles 1 and 6, respectively, with 186 and 110 metabolites had a similar variation at 24 hpi and 96 hpi (**Figure 3G**). Of these profiles, profiles 2 and 7 had significantly more than expected (*P* < 0.05) (**Figure 3H**).

The DAMs between pairwise comparisons were identified in the positive and negative ion models and evaluated by the criteria of VIP ≥1 and *P <*0.05 in the OPLS-DA. As shown in **Supplementary Table 5**, there were 74 (33 up- and 41 downregulated), 91 (47 up- and 44 downregulated), and 87 (56 up- and 31 downregulated) DAMs in both ion models that were screened in the comparison groups CG vs. IG24, CG vs. IG96, and IG24 vs. IG96, respectively. For the top DAMs in different comparisons, orotate, picrotin, N-acetylgalactosamine 6-sulfate, and glaucarubin were upregulated, while L-tryptophan, 3-indoleacrylic acid, adenosine monophosphate, and ornithine were downregulated in the comparison groups CG vs. IG24 (**Supplementary Figure 3A**). Compared with the CG, citric acid, PC (18:1(11Z)/14:0), and methylsuccinic acid were highly increased, while nicotinamide, eicosapentaenoic acid, and docosahexaenoic acid were decreased in the IG96 group (**Supplementary Figure 3B**). In the comparison groups IG24 vs. IG96, L-lysine, ornithine, symmetric dimethylarginine, L-glutamine, and 3-methylxanthine were enhanced, while pyruvic acid, picrotin, salviaflaside methyl ester, and L-lactic acid were inhibited (**Supplementary Figure 3C**).

### 3.4 Functional enrichment of DAMs

**Figure 4:**
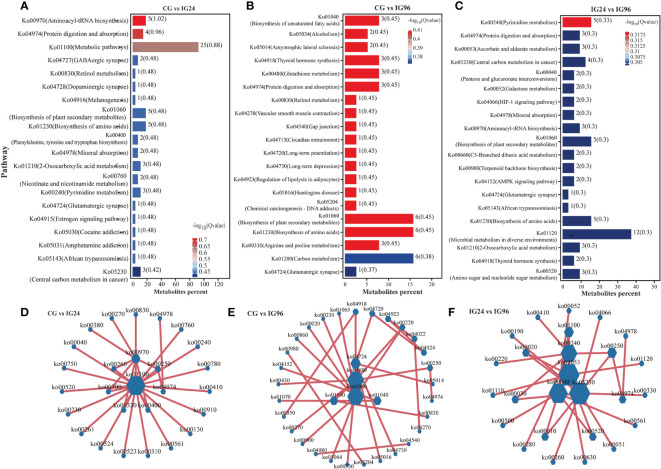
KEGG functional analysis of differential abundance metabolites (DAMs). The top 20 enriched KEGG pathways of DAMs in CG vs. IG24 **(A)**, CG vs. IG96 **(B)**, and IG24 vs. IG96 **(C)**. The numbers beside the column and in the bracket show the number of DAMs and –log_10_(*Q* value), respectively. Interaction network diagram of the KEGG pathway enriched by DAMs in CG vs. IG24 **(D)**, CG vs. IG96 **(E)**, and IG24 vs. IG96 **(F)**. “CG” indicates the control group, and “IG24” and “IG96” indicate the infected groups at 24 and 96 h after *Aeromonas hydrophila* infection.

The KEGG pathway enrichment of DAMs in both positive and negative ion modes was analyzed to identify the metabolic pathways involved in the response to the *A. hydrophila* challenge (**Figure 4**). The results indicated that most DAMs were enriched in aminoacyl-tRNA biosynthesis (Ko00970), protein digestion and absorption (Ko04974), and metabolic pathways (Ko01100) for the comparison groups CG vs. IG24 (**Figure 4A**) as well as in the biosynthesis of amino acids (Ko01230), biosynthesis of plant secondary metabolites (Ko01060), and biosynthesis of unsaturated fatty acids (Ko01040) for the comparison groups CG vs. IG96 (**Figure 4B**). In addition, the top pathways in the comparison groups IG24 vs. IG96 comprised pyrimidine metabolism (Ko00240), microbial metabolism in diverse environments (Ko01120), and biosynthesis of plant secondary metabolites (Ko01060) (**Figure 4C**). Furthermore, the interactions between different pathways enriched by DAMs were used to construct a network illustrating the predominant pathways (**Figures 4D-F**). The core pathways of DAMs were metabolic pathways (Ko01100), glutathione metabolism (Ko00480), and ascorbate and aldarate metabolism (Ko00053) separately in the comparison groups CG vs. IG24 (**Figure 4D**), CG vs. IG96 (**Figure 4E**), and IG24 vs. IG96 (**Figure 4F**).

### 3.5 Integrative analysis of DEGs and DAMs

To identify potential metabolic biomarkers or pathways involved in the response to *A. hydrophila* infection, transcriptomic and metabolomic data were jointly analyzed using three models: an orthogonal partial least squares (O2PLS) model, a functional pathway model, and correlation analysis.

**Figure 5:**
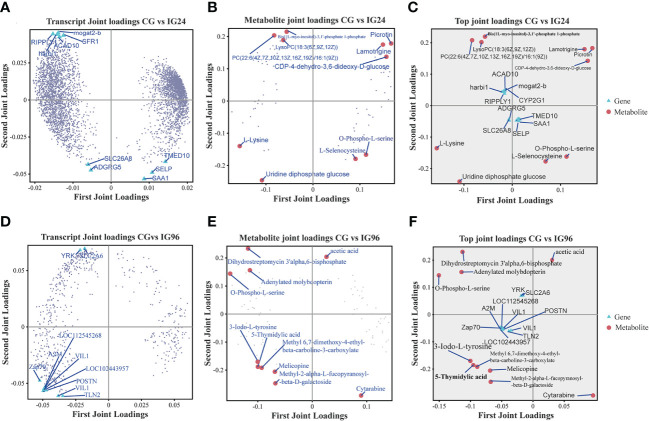
Joint loading plots of DEGs **(A, D)** and DAMs **(B, E)** in different comparisons. The loading plots were analyzed based on the O2PLS model constructed by integrative analysis of DEGs and DAMs. **(A–C)** The transcript loading plots **(A)**, metabolite loading plots **(B)**, and their joint loading plots **(C)** in CG vs. IG24. **(D–F)** The transcript loading plots **(D)**, metabolite loading plots **(E)**, and their joint loading plots **(F)** in CG vs. IG96. DEGs indicate differentially expressed genes. DAMs indicate differentially abundant metabolites. O2PLS indicates orthogonal 2 partial least squares. “CG” indicates the control group, and “IG24” and “IG96” indicate the infected groups at 24 and 96 h after *Aeromonas hydrophila* infection.

The O2PLS model was used to explain the total variation between pairwise comparisons. In this model, joint loading plots of transcriptome and metabolome data were constructed to represent the correlations between metabolites and genes (**Figures 5A-F**). The metabolites highly associated with genes were detected in different comparisons. In the comparison groups CG vs. IG24, the genes *Mogat2*, *Sfr1*, *Ripply*, *harbi1*, *Tmed10*, and *Selp* were highly correlated with the corresponding metabolites, while the metabolites picrotin, lamotrigine, L-lysine, uridine diphosphate glucose, and L-selenocysteine were highly correlated with the corresponding genes (**Figures 5A-C**). In the comparison groups CG vs. IG96, the genes *Yrk*, *Slc2a6*, *Zap70*, *Vil1*, and *Postn* were highly correlated with the corresponding metabolites, while the metabolites acetic acid, dihydrostreptomycin 3′α, 6-bisphosphate, O-phospho-L-serine, 5-thymidylic acid, 3-lodo-L-tyrosine, and cytarabine were closely associated with the related genes (**Figures 5D-F**). In the comparison groups IG24 vs. IG96, the genes *Bysl*, *Apoa1*, *Smc4*, *Cenpe*, and *Foxm1* were highly correlated with the corresponding metabolites, while cytarabine, orotate, furcelleran, lysine, and methylsuccinic acid were closely related to the corresponding genes (**Supplementary Figure 4**).

**Figure 6:**
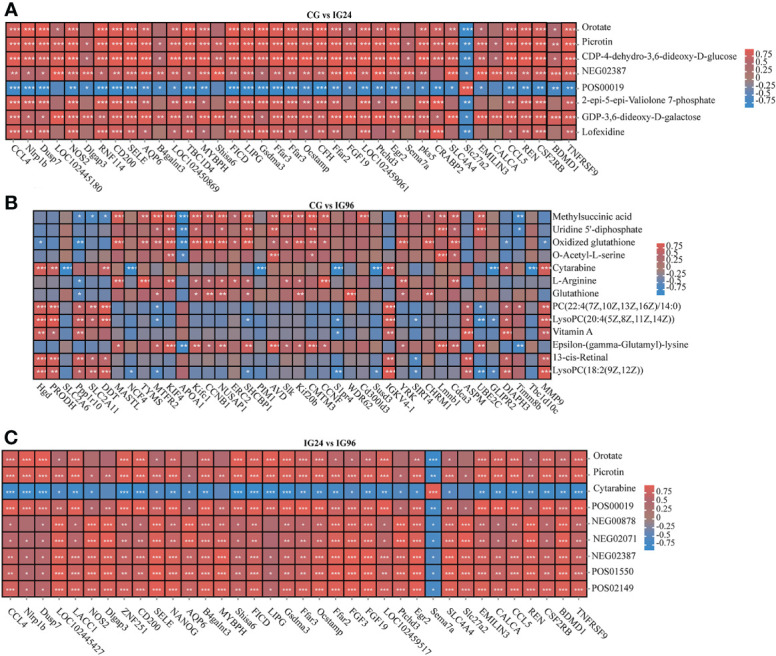
The heat maps of the correlations between DEGs (columns) and DAMs (rows) in CG vs. IG24 **(A)**, CG vs. IG96 **(B)**, and IG24 vs. IG96 **(C)**. The red and blue colors indicate positive and negative correlations between DEGs and DAMs. DEGs indicate differentially expressed genes. DAMs indicate differentially abundant metabolites. “CG” indicates the control group, and “IG24” and “IG96” indicate the infected groups at 24 and 96 h after *Aeromonas hydrophila* infection.

A correlation model was employed to examine the relationships between DEGs and DAMs (**Figure 6**). The correlation matrix for the heatmap shown in the figure represents the positive (red) and negative (blue) associations between the top DEGs and top DAMs that were detected in different pairwise comparisons and evaluated using Pearson correlation coefficients.

**Figure 7:**
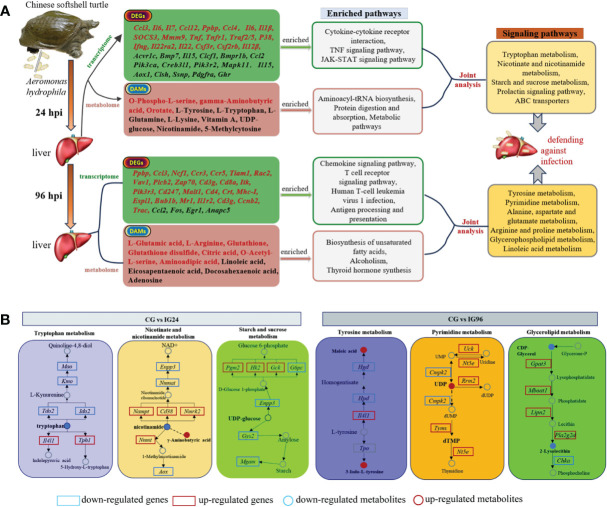
**(A, B)** The summary of core DEGs, DAMs, and enriched pathways in CG vs. IG24 and CG vs. IG96. DEGs indicate differentially expressed genes. DAMs indicate differentially abundant metabolites. “CG” indicates the control group, and “IG24” and “IG96” indicate the infected groups at 24 and 96 h after *Aeromonas hydrophila* infection.

The DEGs and DAMs were further integrated and mapped to the pathway models to search for the crucial signaling pathways of the liver response to the *A. hydrophila* challenge (**Supplementary Table 6**, **Figure 7**). The top pathways at 24 hpi (**Supplementary Table 7**, **Figure 7B**) were involved in the metabolism category, including “tryptophan metabolism,” “retinol metabolism,” nicotinate and nicotinamide metabolism,” “biosynthesis of amino acids,” “starch and sucrose metabolism,” “nitrogen metabolism,” “prolactin signaling pathway,” “ABC transporters,” and “cAMP signaling pathway.” The primary pathways at 96 hpi (**Supplementary Table 7**, **Figure 7**) were “tyrosine metabolism,” “pyrimidine metabolism,” “alanine, aspartate, and glutamate metabolism,” “arginine and proline metabolism,” “glycerophospholipid metabolism,” and “histidine metabolism,” as well as other functional pathways, including “thyroid hormone” and “FoxO signaling pathway.” The differential pathways between 24 hpi and 96 hpi were related to metabolism, signal transduction, and the endocrine system (**Supplementary Table 7**).

### 3.6 Validation of RNA-seq results

Fourteen genes involved in immunity and metabolism were randomly chosen to validate the transcriptomic data (**Supplementary Table 1**, **Supplementary Figure 5**). These genes included *Tlr3* (toll-like receptor 3), *Tlr4* (toll-like receptor 4), *Tlr5* (toll-like receptor 5), *Tlr8* (toll-like receptor 8), *Ccl3* (C-C motif chemokine 3-like), *Mapk11* (mitogen-activated protein kinase 11), *Ap-1* (Fos proto-oncogene, AP-1 transcription factor subunit), *Tph1* (tryptophan hydroxylase 1), *Tdo2* (tryptophan 2,3-dioxygenase), *Kmo* (kynurenine 3-monooxygenase), *Anapc5* (anaphase-promoting complex subunit 5), *Dck* (deoxycytidine kinase), *Tyms* (thymidylate synthetase), and *Cmpk2* (cytidine/uridine monophosphate kinase 2). The fold change of RT-PCR results was different from the RNA-seq results. However, the *R* ^2^ values of Pearson correlation coefficients between the RT-PCR and transcriptomic results were 0.94 and 0.86, respectively, in the comparison groups CG vs. IG24 and CG vs. IG96, indicating that the mRNA levels of these genes were in agreement with the transcriptomic results. These results confirmed the accuracy and reliability of the transcriptomic data.

## 4 Discussion

In the intensive culture environment, Chinese soft-shelled turtles are susceptible to *A. hydrophila* infection. Previous papers have studied the immune mechanism of Chinese soft-shelled turtles in resisting *A. hydrophila* challenge at the mRNA expression level (6). There is little research exploring the metabolic pattern of Chinese soft-shelled turtles during *A. hydrophila* infection. In the current study, we combined the transcriptome and metabolome to find the key metabolites and the signaling pathways of Chinese soft-shelled turtle’s liver in dealing with *A. hydrophila* infection at different infective stages.

### 4.1 Plasma biochemical indices changed after infection

The detection of GPT, GOT, and AKP activities is a common way to diagnose damage to the liver (41). GPT and GOT, as aminotransferases, can catalyze the redistribution of nitrogen between amino acids and corresponding oxoacids, thereby regulating protein metabolism and gluconeogenesis (20). In our study, plasma GPT and GOT activities were significantly increased to the highest levels at 24 h after *A. hydrophila* infection and decreased gradually at the later infective stages, indicating that *A. hydrophila* infection led to severe hepatic damage at the initial stage of infection. Similarly, GPT and GOT activities were elevated in Japanese flounder in response to heat stress (20) and in crucian carp after *A. veronii* challenge (21).

The plasma AKP activities were increased from 12 hpi to 48 hpi and then decreased to the initial levels at 96 hpi compared with the control group. The observed AKP activity may indicate that the Chinese soft-shelled turtles exhibited a stronger immune response at the earlier stages from 12 h to 48 h after *A. hydrophila* infection, while the indicators returned to their initial levels at 96 hpi. Previous studies have found that aquatic animal AKP activities can be improved in response to either nutritive or environmental stress (42). Plasma AKP activity in the Chinese mitten crab was enhanced after *A. hydrophila* infection (21). Furthermore, *A. veronii* challenge increased plasma AKP activity in loaches (43).

The liver is the major organ responsible for endogenous glucose production, especially via gluconeogenesis and glycogenolysis. Glucose is metabolized into pyruvate through glycolysis in the cytoplasm, and pyruvate is completely oxidized to generate ATP through the TCA cycle and oxidative phosphorylation in the mitochondria. In the fed state, glycolytic products are used to synthesize fatty acids through *de-novo* lipogenesis (44). Blood glucose, involved in energy regulation, is a sensitive indicator of various stressors in aquatic animals (45). In this study, plasma glucose concentrations of the Chinese soft-shelled turtle reached the highest levels at 24 hpi and returned to relatively high levels from 48 hpi to 96 hpi compared with the control group. The increased plasma glucose after *A. hydrophila* infection could be attributed to glycogen mobilization into glucose to resist the bacterial challenge via enhanced glycogenolysis and the diminished glycolytic pathway (45).

In the current research, plasma CAT activity and MDA content were remarkably elevated at 24 hpi compared with the control group and then declined to their initial levels. Similarly, CAT activities were increased in Nile tilapia injected with a ghost vaccine *Streptococcus agalactiae* (46), and MDA was elevated in crucian carp following *A. hydrophila* infection (47). Our findings indicated that Chinese soft-shelled turtles may improve CAT activity to eliminate ROS at 24 hpi. However, the accumulated ROS at 24 hpi could be beyond the threshold of CAT, leading to an increase in MDA content induced by ROS reacting with membrane lipids.

### 4.2 Key genes responding to *Aeromonas hydrophila* infection

The innate immune system is the first line of defense against pathogens. The system rapidly detects and destroys pathogens compared with acquired immunity. The synthesis of cytokines and chemokines elicits diverse inflammatory responses that recruit macrophages and neutrophils to the site of inflammation (48). In this study, TNF signaling pathways showed higher mRNA expression at 24 hpi, as shown by the levels of *Tnf*, *Tnfr1*, *Traf2*, *Traf3*, *Traf5*, *Tnfr1*, *Ciap1/2*, *Rip1*, and *Rip3*. Abundant genes of the CC subfamily, such as the *Ccl3*, *Ccl3l1*, *Ccl4*, *Ccl4l1*, *Ccl4l2*, *Ccl5*, *Ccl17*, *Ccl20*, *Ccl22*, *Ccr5*, and *Ccr11*, as well as the CXC subfamily, comprising *Cxcl1*, *Cxcl2*, *Cxcl3*, *Cxcl10*, *Cxcl11*, *Cxcl13*, *Cxcl14*, and *Cxcr2*, were upregulated at 24 hpi. Moreover, *Ccl4*, *Ccl4l1*, *Ccl4l2*, *Ccr5*, *Ccl20*, and *Cxcl13* were upregulated at 96 hpi. These results indicated that innate immunity, including TNF signaling pathways inducing inflammation and chemokines triggering the migration of immune cells, may be the predominant mechanisms employed to clear *A. hydrophila* at the early stages of infection in Chinese soft-shelled turtles. Similar research demonstrated that *ccl34a.4* expression was increased 24 h after bacterial infection in zebrafish (49).

After pathogen recognition, activated T-cell receptors in cooperation with signaling pathways can trigger adaptive immunity by driving the differentiation of activated T cells to specific T-cell subtypes (50). Our research found that the DEGs associated with the T-cell receptor were specifically upregulated at 96 hpi, including *Cd45*, *Cd4/8*, *Cd3δ*, *Cd3ζ*, *Tcrα*, *Tcrβ*, and *Zap70*. The DEGs at different infective stages implied that the liver resisted the *A. hydrophila* infection via innate immunity by improving cytokine mRNA expression at the early infective stages (24 hpi), and the response switched to adaptive immunity by triggering T-cell receptors at the later infective stages (96 hpi).

### 4.3 Key metabolites responding to *Aeromonas hydrophila* infection

In the current research, 74 and 91 DAMs were detected at 24 and 96 hpi, respectively, compared with the control group. Orotate, picrotin, N-acetylgalactosamine 6-sulfate, and glaucarubin were upregulated, while L-tryptophan, 3-indoleacrylic acid, adenosine monophosphate, and ornithine were downregulated metabolites 24 h after *A. hydrophila* infection. Orotate is a precursor in the biosynthesis of pyrimidines, compounds that play important roles in cellular apoptosis inhibition, antioxidation, and anti-inflammatory activity (51). Tryptophan, acting as a neurotransmitter and inhibiting the activities of inflammatory cytokines, can maintain immune homeostasis by regulating T-lymphocyte-mediated immunity (52). Ornithine, catalyzed by ornithine decarboxylase and S-adenosylmethionine decarboxylase, is converted to polyamines in M2 macrophages and has important functions in infection- or injury-induced tissue healing. Similarly, L-tryptophan and adenosine monophosphate were decreased in mud crabs infected by *Vibrio parahaemolyticus* (53). Supplementation of ornithine in rainbow trout can alter the mRNA expression of hepatic immune genes following *Aeromonas salmonicida* infection (54). Our results indicated that the metabolic processes of Chinese soft-shelled turtles were significantly affected 24 h after *A. hydrophila* infection.

Multiple organic acids were significantly altered at 96 hpi in this research. For example, citric acid and methylsuccinic acid were increased, while eicosapentaenoic acid and docosahexaenoic acid were decreased. Citric acid, as the key substance in the TCA cycle, is an intermediate connecting the metabolism of carbohydrates and fatty acids, processes that can further facilitate the proliferation and differentiation of immune cells, such as B cells (55). A previous study found that 2%–3% dietary citric acid could improve the immunity of Japanese quail (56). Nicotinamide, also known as vitamin B_3_, is synthesized from tryptophan and is converted into nicotinamide adenine dinucleotide (NAD^+^) (57). It has been demonstrated that nicotinamide can enhance innate immunity at dosages up to 1,000-fold of the normal level, thus having potential application in the resistance against pathogens. For example, nicotinamide can protect the mud crab against *Staphylococcus aureus* infections (53). Nicotinic acid supplementation can reduce inflammation in monocytes of atherosclerosis models (58). Here, nicotinamide was significantly reduced at 96 hpi, indicating that the nicotinamide-mediated immunomodulatory functions might be inhibited at the later stages of the *A. hydrophila* challenge. Similar results in *Nibea albiflora* showed that nicotinamide was downregulated at 24 h and upregulated at 72 h after *Cryptocaryon irritans* infection (27). Moreover, the amounts of DAMs varied between 24 and 96 hpi in this research, implying that different hepatic metabolites were involved in defending against *A. hydrophila* challenge in a time-dependent manner in Chinese soft-shelled turtles.

### 4.4 Crucial signaling pathways involved in resisting *Aeromonas hydrophila* infection

An integrative analysis of DEGs from the transcriptome and DAMs from the metabolome was performed to investigate the hepatic signaling pathways involved in the response against *A. hydrophila* challenge in Chinese soft-shelled turtles. The main pathways against bacterial infection were classified into amino acid metabolism, nucleotide metabolism, metabolism of cofactors and vitamins, and energy metabolism.

Amino acid metabolism has crucial functions in diverse metabolic processes, including protein synthesis, ATP generation, and nucleotide synthesis. Amino acid metabolism has a profound influence on the functions of immune cells (59). For example, tryptophan is an essential component of immune cell metabolism and T-cell proliferation (59). Glutamine is considered the “fuel for the immune system”, as it can promote lymphocyte proliferation, cytokine production, and neutrophil bacterial killing (60). In our results, L-glutamic acid was decreased at 24 hpi but increased at 96 hpi. The change in L-glutamic acid triggered nitrogen metabolism and central carbon metabolism in cancer at 24 hpi. Afterward, alanine, aspartate, and glutamate metabolism; arginine and proline metabolism; and histidine metabolism were activated at 96 hpi. These results suggested that amino acid metabolism played a vital function in Chinese soft-shelled turtles’ resistance to bacterial infection. Similarly, histidine metabolism is involved in dealing with acute nitrite stress in Chinese soft-shelled turtles (61). The amino acid metabolism in the liver is a pivotal pathway for Yangtze sturgeon to resist heat stress (30).

Previous studies have found that nicotinate and nicotinamide metabolism (62) as well as pyrimidine metabolism (63) exerts anti-inflammatory effects. Nicotinamide, as the core component of nicotinate and nicotinamide metabolism, can reduce oxidative stress and inflammation by regulating the energy metabolism of cells (57). Pyrimidine nucleotides are precursors for activated carbohydrates and lipids. Abnormality of purine metabolism results in a deficiency of immune function (64). In the current study, nicotinate and nicotinamide metabolism at 24 hpi and pyrimidine metabolism at 96 hpi were enriched in the liver, indicating that hepatic DNA synthesis was affected in Chinese soft-shelled turtles after *A. hydrophila* challenge. A previous study showed that some metabolites involved in pyrimidine metabolism were influenced in the mud cab after *V. parahaemolyticus* infection (53); nicotinate and nicotinamide metabolism was modulated in *N. albiflora* challenged by *C. irritans* (27).

Energy metabolism is a crucial biological process for organism survival. Sucrose metabolism plays a crucial part in development and stress response. A range of sugars can be used as fuel to promote growth and synthesize essential compounds (including proteins, cellulose, and starch). Sugars also can be considered signals to regulate the expression of functional genes associated with hormonal, oxidative, and defense signaling (65). Nitrogen metabolism has important functions in clearing excess nitrogen from the body when amino acids are converted to energy (66). Our research found that starch and sucrose metabolism as well as nitrogen metabolism was enriched at 24 h after *A. hydrophila* infection, indicating that starch and sucrose metabolism could be employed to produce energy at the early stages of *A. hydrophila* infection in Chinese soft-shelled turtles. Similarly, starch and sucrose metabolism and nitrogen metabolism are regulated in *Pelteobagrus fulvidraco* after *A. veronii* infection (29).

Lipid metabolism can regulate the immune response of aquatic animals to adverse stimuli by providing more energy-yielding nutrients (67). Glycerophospholipids are one of the most abundant phospholipids in vertebrates and have been demonstrated to be involved in both general systemic-immune and low-grade inflammatory states, indicating a potential role in immunity modulation (68). The glycerophospholipid metabolism and linoleic acid metabolism of *N. albiflora* can respond to *C. irritans* infection (29). In this study, hepatic glycerophospholipid metabolism and linoleic acid were altered at 96 h after *A. hydrophila* infection, in contrast to glycometabolism at 24 hpi. These data indicate that the liver tissue may initially mobilize glycometabolism to generate energy for resistance against bacteria at the early infective stages and then switch to lipid metabolism to supply energy at the later infective stages in Chinese soft-shelled turtles.

## 5 Conclusions

This study explored the temporal patterns of plasma biochemical indices and liver metabolic and transcriptomic variation in Chinese soft-shelled turtles infected with *A. hydrophila* to systemically characterize the core molecular markers and metabolites involved in the response against infection. The association between the genes and the metabolites further focused on the important signaling pathways against bacterial challenge in a time-dependent manner. The screened metabolites and signaling pathways may provide valuable resources for future studies on bacterial disease prevention in Chinese soft-shelled turtles.

## Data availability statement

The datasets presented in this study can be found in online repositories. The names of the repository/repositories and accession number(s) can be found in the article/**Supplementary Material**.

## Ethics statement

The animal study was approved by Ethics Committee of the Pearl River Fisheries Research Institute, Chinese Academy of Fishery Sciences. The study was conducted in accordance with the local legislation and institutional requirements.

## Author contributions

LJ: Conceptualization, Investigation, Project administration, Validation, Writing – original draft, Writing – review & editing. CC: Investigation, Project administration, Writing – review & editing. JZ: Project administration, Writing – review & editing. XH: Methodology, Writing – review & editing. XL: Methodology, Writing – review & editing. CW: Project administration, Writing – review & editing. XZ: Funding acquisition, Supervision, Writing – review & editing. WL: Funding acquisition, Writing – review & editing.

## Supplementary material

Supplementary Figure 1 Global analysis of differentially expressed genes (DEGs). **(A)** The correlation matrix of gene expression among five biological triplicates of different groups (n = 5). **(B)** PCA plot showing the variations of gene expression profiles in three groups. **(C)** Venn diagram analysis of DEGs in CG vs IG24, CG vs IG96, and IG24 vs IG96. **(D)** Number of DEGs (|log2(FoldChange)| > 1 and adjusted *P*-value < 0.05) in CG vs IG24, CG vs IG96, and IG24 vs IG96. Red and blue indicated up- and down-regulated genes, respectively. “CG” indicated the control group, “IG24”, and “IG96” respectively indicated the infected groups at 24 and 96 hours post *A. hydrophila* infection.

Supplementary Figure 2 Global analysis of differential abundance metabolites (DAMs). **(A)** Correlation analysis between biological replicates in three groups. **(B)** Principal component analysis (PCA) of metabolites at 0, 24, and 96 hours post infection. PLS-DA score plots and cross-validation results in CG vs IG24 **(C)**, CG vs IG96 **(D)**, and IG24 vs IG96 **(E)**. “R2Y” indicated the explanatory rate of the PLS-DA model, “Q2Y” indicated the predictive ability of this model. “CG” indicated the control group, “IG24”, and “IG96” indicated the infected groups on 24 and 96 hours post *A. hydrophila* infection.

Supplementary Figure 3 The z-score analysis showed the top 20 differential expressed metabolites in CG vs IG24 **(A)**, CG vs IG96 **(B)**, and IG24 vs IG96 **(C)**. “CG” indicated the control group, “IG24”, and “IG96” indicated the infected groups at 24 and 96 hours post *A. hydrophila* infection.

Supplementary Figure 4 The loading plots of DEGs **(A)**, DAMs **(B)** and their joint loading plots **(C)** in IG24 vs IG96. The loading plots were analyzed base on the O2PLS model constructed by integrative analysis of DEGs and DAMs. DEGs indicated differential expressed genes. DAMs indicated differential abundance metabolites. O2PLS indicated orthogonal 2 partial least squares. “CG” indicated the control group, “IG24”, and “IG96” indicated the infected groups at 24 and 96 hours post *A. hydrophila* infection.

Supplementary Figure 5 Pearson correlation analysis of hepatic mRNA levels between real-time PCR and RNA-Seq result in CG vs IG24 **(A)** and CG vs IG96 **(B)**. “CG” indicated the control group, “IG24”, and “IG96” indicated the infected groups at 24 and 96 hours post *A. hydrophila* infection.
