# Supplementary material for: Integrated time-series biochemical, transcriptomic, and metabolomic analyses reveal key metabolites and signaling pathways in the liver of the Chinese soft-shelled turtle (Pelodiscus sinensis) against Aeromonas hydrophila infection
Source: Front Immunol. 2024 May 10;15:1376860. doi: 10.3389/fimmu.2024.1376860 (PMC11116567; doi:10.3389/fimmu.2024.1376860)
Supplement: Supplementary file 2 [file Table_2.docx]

**Table S2 Overview of the sequencing quality of transcriptome**

| Sample | Raw Reads | CR | CR Ratio (%) | Q20 (%) | Q30 (%) | GC Content (%) | TMG (%) |
| --- | --- | --- | --- | --- | --- | --- | --- |
| CG1 | 43280994 | 43060596 | 99.49 | 98.09 | 94.28 | 46.51 | 88.97 |
| CG2 | 55834210 | 55573058 | 99.53 | 98.38 | 94.78 | 45.55 | 90.81 |
| CG3 | 48982094 | 48753868 | 99.53 | 98.22 | 94.47 | 45.26 | 89.99 |
| CG4 | 47408758 | 47055384 | 99.25 | 97.89 | 93.70 | 46.09 | 88.58 |
| CG5 | 57071244 | 56930026 | 99.75 | 98.18 | 94.48 | 47.05 | 88.42 |
| IG24-1 | 43117692 | 42930812 | 99.57 | 98.03 | 94.03 | 45.94 | 89.51 |
| IG24-2 | 48758654 | 48533562 | 99.54 | 98.02 | 94.02 | 46.58 | 88.67 |
| IG24-3 | 47340610 | 47158186 | 99.61 | 97.97 | 93.96 | 46.30 | 88.25 |
| IG24-4 | 45212188 | 44988258 | 99.50 | 98.14 | 94.43 | 46.23 | 88.89 |
| IG24-5 | 39249030 | 39040570 | 99.47 | 97.89 | 93.80 | 47.34 | 87.64 |
| IG96-1 | 42710996 | 42513954 | 99.54 | 98.21 | 94.49 | 46.86 | 88.85 |
| IG96-2 | 46166372 | 45905848 | 99.44 | 97.83 | 93.52 | 45.73 | 89.92 |
| IG96-3 | 51933494 | 51933494 | 99.48 | 97.59 | 93.03 | 46.29 | 88.31 |
| IG96-4 | 42184080 | 41875918 | 99.27 | 97.86 | 93.57 | 46.22 | 88.88 |
| IG96-5 | 57572662 | 57265530 | 99.47 | 98.26 | 94.74 | 46.01 | 89.74 |

For convenience, “CG”indicated the control group, “IG24” and “IG96” indicated the infected groups on 24 and 96 hours post *A. hydrophila* infection. “CR” indicated clean reads. “TMG” indicated the ratio of the total clean reads mapped to genome.
