# Supplementary material for: Integrated time-series biochemical, transcriptomic, and metabolomic analyses reveal key metabolites and signaling pathways in the liver of the Chinese soft-shelled turtle (Pelodiscus sinensis) against Aeromonas hydrophila infection
Source: Front Immunol. 2024 May 10;15:1376860. doi: 10.3389/fimmu.2024.1376860 (PMC11116567; doi:10.3389/fimmu.2024.1376860)
Supplement: Supplementary file 5 [file Table_5.docx]

**Table S5** Statistics of differential abundance metabolites in pairwise comparison. “CG”indicated the control group, “IG24”, and “IG96” indicated the infected groups on 24 and 96 hours post *A. hydrophila* infection.

| Groups | Positive ion model | | | Negative ion model | | | Two models | | |
| --- | --- | --- | --- | --- | --- | --- | --- | --- | --- |
|  | Up | Down | Total | Up | Down | Total | Up | Down | Total |
| CG vs IG24 | 12 | 25 | 37 | 21 | 16 | 37 | 33 | 41 | 74 |
| CG vs IG96 | 21 | 21 | 42 | 27 | 24 | 51 | 47 | 44 | 91 |
| IG24 vs IG96 | 45 | 10 | 55 | 10 | 22 | 32 | 56 | 31 | 87 |
