# Supplementary material for: Integrated time-series biochemical, transcriptomic, and metabolomic analyses reveal key metabolites and signaling pathways in the liver of the Chinese soft-shelled turtle (Pelodiscus sinensis) against Aeromonas hydrophila infection
Source: Front Immunol. 2024 May 10;15:1376860. doi: 10.3389/fimmu.2024.1376860 (PMC11116567; doi:10.3389/fimmu.2024.1376860)
Supplement: Supplementary file 7 [file Table_7.docx]

Table S7 The top 10 pathways enriched by integration of differentially expressed genes and different abundance metabolites

| CG vs IG24 | | | CG vs IG96 | | |
| --- | --- | --- | --- | --- | --- |
| Pathway | DAMs | DEGs | Pathway | DAMs | DEGs |
| Tryptophan metabolism | 1. Tryptophan | *Aadat; Acat2; Haao; Kynu; Cyp1B1; Tdo2; Aox1; Kmo; Ido2; Aanat; Acmsd; Cyp1A5; Cyp1A5; Maob; Cat; Tph1; Il4i1; Aadat; Cyp1A1* | Tyrosine metabolism | Maleic acid | *Hgd; Hpd; Comt; Il4i1* |
| Prolactin signaling pathway | 1. Tyrosine | *Socs1; Tnfsf11; Tnfrsf11A; Socs3; Irf1; Cish; Slc2A2; Socs2; Fos; Slc2A2; Foxo3; Esr1; Nfkb1; Pik3R3; Slc2A2; Gck; Rela; Pik3r2; Mapk11; Pik3Ca; Shc2; Grap; Src; Ccnd2; Stat1; Socs7* | Pyrimidine metabolism | 5-Thymidylic acid; Uridine 5'-diphosphate | *Dck;* *Nt5e; Cmpk2; Tyms; Rrm2; Uck2* |
| ABC transporters | 1. Glutamine; L-Lysine | *Abcc12; Abca13; Abcg8; Abcg2; Abcg5; Abcb1; Abcg1; Abca4; Abcd2; Abcb11; Abcb9; Abca5; Abcc1; Abca9; Abca8; Abcc2* | Thyroid hormone synthesis | Glutathione; Oxidized glutathione | *Adcy7; Pdia4; Hsp90B1; Slc26A4; Plcb2; Gpx7* |
| Retinol metabolism | Vitamin A | *Ugt1A1; Dhrs3; Aldh1A2; Dgat1; Cyp26b1; Retsat; Adh1; Aox1; Hsd17B6; Cyp1A5; Bco1; Rpe65; Cyp2C3; Rdh10; Cyp1A1* | Alanine, aspartate and glutamate metabolism | L-Glutamic acid; Citric acid | *Aspa; Il4i1; Asrgl1* |
| Nicotinate and nicotinamide metabolism | Nicotinamide;gamma-Aminobutyric acid | *Nmnat3; Nt5E; Nadk2; Cd38; Nt5c1b;* *Enpp3; Nnmt; Aspdh; Aox1; Sirt3; Nampt; Qprt; Sirt4;* *Nmrk2* | Arginine and proline metabolism | L-Glutamic acid | *Prodh; Cndp1; Nos2; Sat2* |
| cAMP signaling pathway | γ-Aminobutyric acid | *Rap1B; Rapgef4; Vav2; Ptch1; Creb3L1; Pln; Tiam1; Creb3L2; Fshr; Cngb1; Cacna1D; Plce1; Atp2B2; Sucnr1; Adcy6; Adcy8; Sstr2; Ppara; Gnai1; Pld1; Pld2; Afdn; Fos; Jun; Pik3R3**; Rac2; Mc1r* | FoxO signaling pathway | L-Glutamic acid | *Plk1; Ccnb1; Ccnb2; S1pr4; Pik3R3; Prkag2* |
| Central carbon metabolism in cancer | L-Tyrosine; L-Tryptophan; L-Glutamine | *Pdgfra; Fgfr2; Fgfr3; Met; Hk2; Pfkm; Slc2A2; Slc7A5; Slc2A2; Slc1A5; Ntrk1; Pkm; Sirt3; Pik3R3; Slc2A2; Gck; Pik3r2; Pik3Ca; Pdk1* | Glycerophospholipid metabolism | LysoPC(18:3(6Z,9Z,12Z)) | *Gpat3; Mboat1; Pla2G4C;* *Pla2g2d; Chka; Lpin2* |
| Biosynthesis of amino acids | L-Tyrosine; L-Tryptophan; L-Glutamine; L-Lysine | *Glul; Shmt1; Nags; Tkt; Tat; Aldob; Pfkm; Arg1; Glul; Psat1; Prps1; Pah; Mat1A; Bcat1; Pkm; Asns; Cps1; Cps1* | Histidine metabolism | L-Glutamic acid | *Aspa; Cndp1* |
| Starch and sucrose metabolism | Uridine diphosphate glucose | *G6Pc; G6Pc; Hk2; Enpp3; Gys2; Enpp3; Gba3; Mgam; Gck; Pgm2; G6Pc3; Gaa* | Linoleic acid metabolism | Linoleic acid | *Pla2G4C; Pla2g2d; Cyp2J2* |
| Nitrogen metabolism | L-Glutamine | *Glul; Glul; Ca3; Ca4; Ca6; Cps1; Cps1* | Glutamatergic synapse | L-Glutamic acid | *Pla2G4C; Adcy7; Grk3; Slc1A3; Plcb2* |

| IG24 vs IG96 | | | | | | |
| --- | --- | --- | --- | --- | --- | --- |
| Pathway | | DAMs | | DEGs | | |
| Insulin resistance | | Pyruvic acid | | *G6Pc; Ppargc1A; Rps6ka3; Tnfrsf1A; Creb3L1; Creb3L2; Socs3; Cpt1A; Gys2; Tnf; Foxo1; Il6; Irs2; Slc2A2; Ppara; Prkcd; Ppp1r3cb; Cd36; Mlxipl; Slc2A2; Irs1; Gfpt2; Nfkb1; Pik3R3; Rela; Rps6kb1; Prkaa2; Trib3; Nfkbia; Slc27a2; Prkag2; Pik3r2; Pik3Ca; Pck1; Prkab1; Pck2; G6Pc3; Irs2* | | |
| AMPK signaling pathway | | ADP; Pyruvic acid | | *G6Pc; Ppargc1A; Eef2K; Pfkfb3; Creb3L1; Pfkfb2; Creb3L2; Tsc2; Pfkm; Ppp2R2A; Cpt1A; Gys2; Ppp2R2D; Foxo1; Adipor1; Irs2; Scd1; Cd36; Ppp2r5b; Rab10; Irs1; Foxo3; Pik3R3; Ppp2R2B; Rps6kb1; Prkaa2; Pparg; Ppp2R2C; Prkag2; Pik3r2; Pik3Ca; Pck1; Prkab1; Pck2; G6Pc3; Irs2* | | |
| Glucagon signaling pathway | | L-Lactic acid; Pyruvic acid | | *G6Pc; Ppargc1A; Creb3L1; Creb3L2; Gnas; Pfkm; Pdhb; Cpt1A; Gys2; Plcb3; Atf4; Foxo1; Slc2A2; Ppara; Sik2; Slc2A2; Plcb4; Gck; Ppp3Ca; Gnas; Prkaa2; Acacb; Prkag2; Itpr1; Pck1; Prkab1; Pck2; G6Pc3* | | |
| Tryptophan metabolism | | L-Tryptophan | | *Aadat; Acat2; Aldh16A1; Haao; Kynu; Cyp1B1; Tdo2; Aox1; Kmo; Ido2; Acmsd; Cyp1A5; Cyp1A5; Maob; Cat; Il4i1* | | |
| FoxO signaling pathway | | ADP | | *G6Pc; Plk1; Gadd45G; Foxo1; Il7R; Il6; Bcl6; Cdkn1A; Irs2; Plk3; Il10; Irs1; Foxo3; S1pr4; Gadd45A; Pik3R3; Tgfbr2; Raf1; Prkaa2; Cat; Cdk2; Tnfsf10; Gadd45B; Prkag2; Pik3r2; Homer2; Mapk11; Pik3Ca; Ccnd2; Pck1; Prkab1; Sgk2; Pck2; G6Pc3* | | |
| Type II diabetes mellitus | | Pyruvic acid | | *Socs1; Socs3; Hk2; Cacna1D; Tnf; Irs2; Slc2A2; Prkcd; Socs2; Slc2A2; Abcc8; Irs1; Pik3R3; Gck; Pik3r2; Pik3Ca; Irs2* | | |
| Biosynthesis of amino acids | | O-Acetyl-L-serine; L-Tryptophan; L-Glutamine; L-Lysine; Pyruvic acid | | *Glul; Shmt1; Tat; Aldob; Pfkm; Eno4; Arg1; Glul; Mat2A; Psat1; Prps1; Sds; Pycr2; Mat1A; Bcat1; Idh1; Asns; Cps1; Cps1* | | |
| Inositol phosphate metabolism | | myo-Inositol | | *Plce1; Impa1; Pik3C2B; Plcb3; Itpka; Ippk; Mtmr7; Itpk1; Plch2; Inpp4B; Plcd3; Plcb4; Pip4K2C; Pip5K1B; Pip4K2B; Plcg1; Synj2; Inpp4A; Inpp5D; Pik3Ca; Plce1; Plch1; Plcg1* | | |
| cAMP signaling pathway | | L-Lactic acid | | *Rapgef4; Vav2; Ptch1; Gcgr; Creb3L1; Pln; Creb3L2; Gnas; Cngb1; Cacna1D; Plce1; Atp2B2; Sucnr1; Adcy6; Adcy8; Adcy7; Fxyd1; Ptger3; Ppara; Nfatc1; Gnai1; Pld1; Pld2; Afdn; Fos; Ffar3; Ffar3; Ffar2; Nfkb1; Pak1; Pik3R3; Rela; Pde4B; Raf1; Vav3; Npy1R; Gnas; Hhip; Pde3A; Atp1A1; Nfkbia; Atp2A2; Pik3r2; Vipr2; Vav1; Pik3Ca; Sstr1; Adcy10; Plce1; Vav1* | | |
| *Phosphatidylinositol signaling system* | | *myo-Inositol* | | *Plce1; Impa1; Pik3C2B; Plcb3; Itpka; Ippk; Mtmr7; Itpk1; Ip6K2; Inpp4B; Plcd3; Plcb4; Pik3R3; Pip4K2C; Pip5K1B; Pip4K2B; Plcg1; Synj2; Ppip5K2; Inpp4A; Pik3r2; Inpp5D; Pik3Ca; Itpr1; Plce1; Dgkd; Itpkb* | | |
|  | |  | |  |  |  |
