# Supplementary material for: Integrated time-series biochemical, transcriptomic, and metabolomic analyses reveal key metabolites and signaling pathways in the liver of the Chinese soft-shelled turtle (Pelodiscus sinensis) against Aeromonas hydrophila infection
Source: Front Immunol. 2024 May 10;15:1376860. doi: 10.3389/fimmu.2024.1376860 (PMC11116567; doi:10.3389/fimmu.2024.1376860)

Supplementary Figure

Supplementary Figure S1

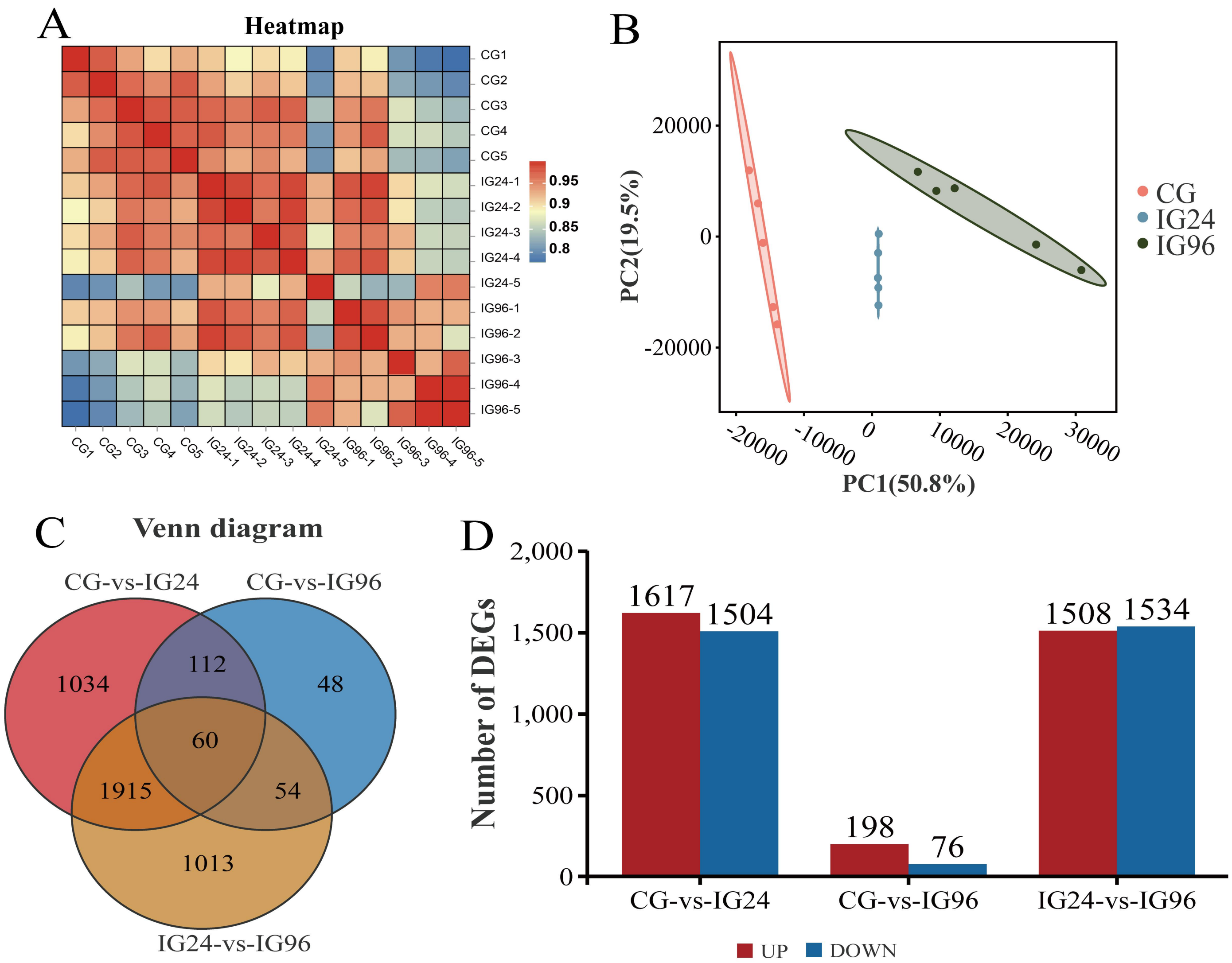

Supplementary Figure S2

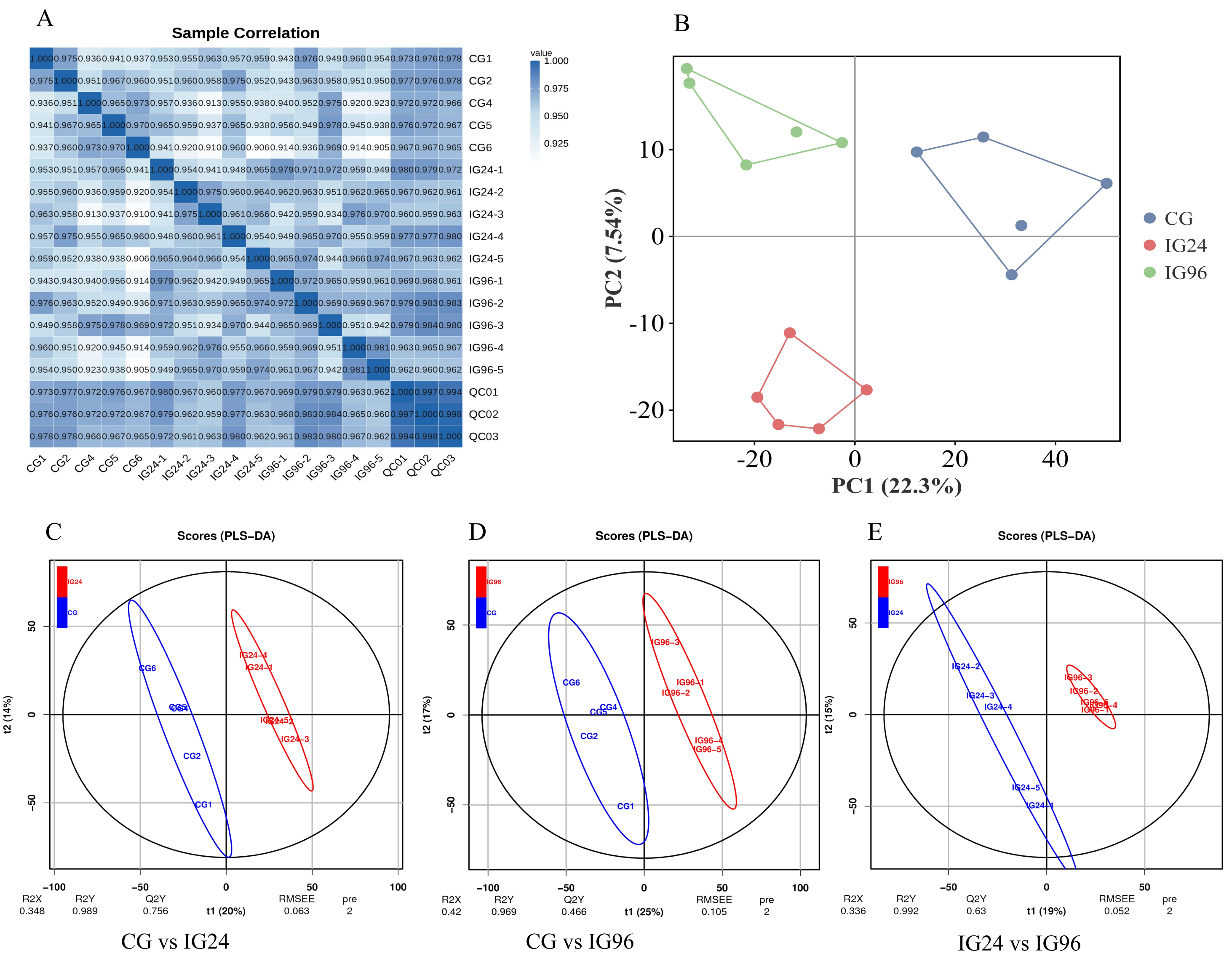

Supplementary Figure S3

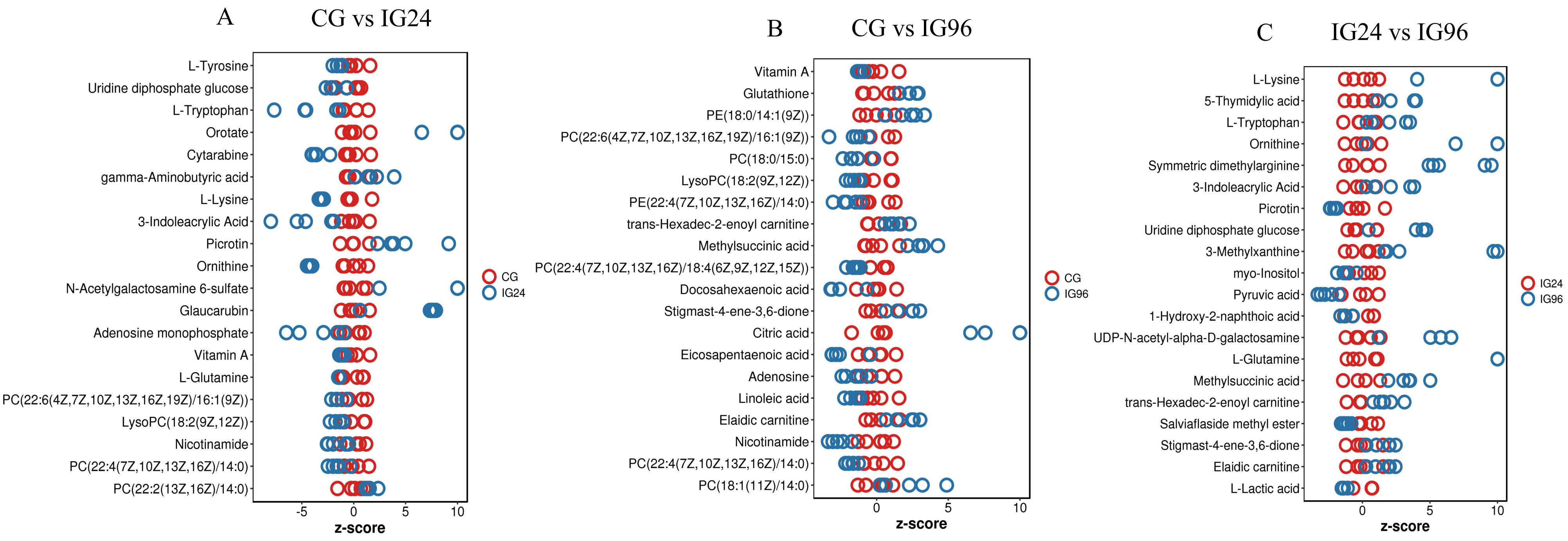

Supplementary Figure S4

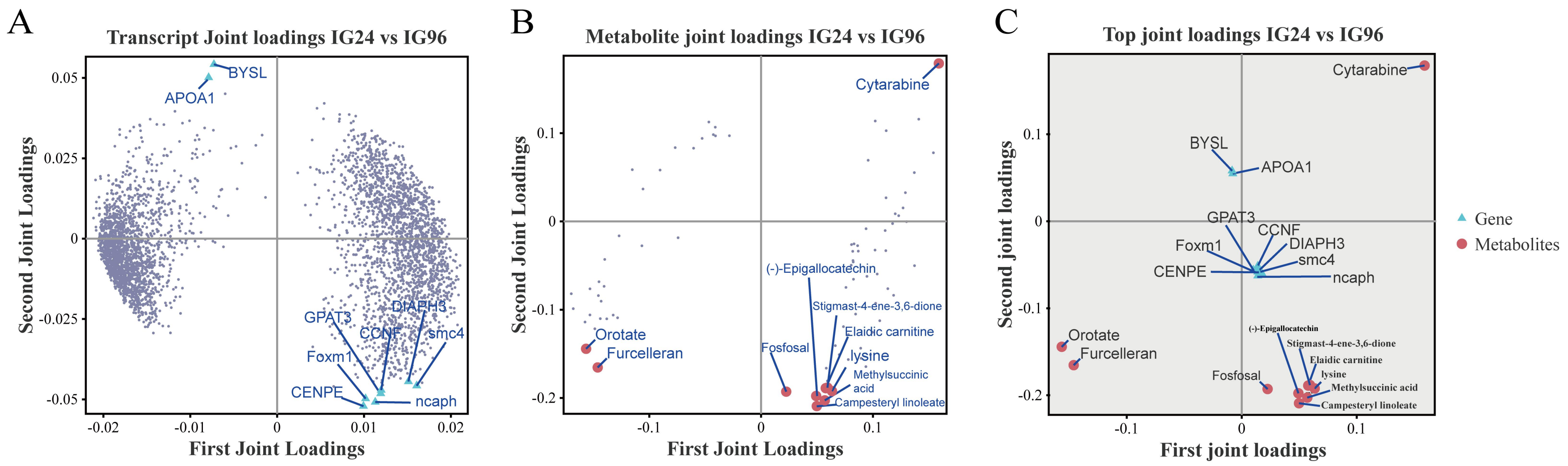

Supplementary Figure S5

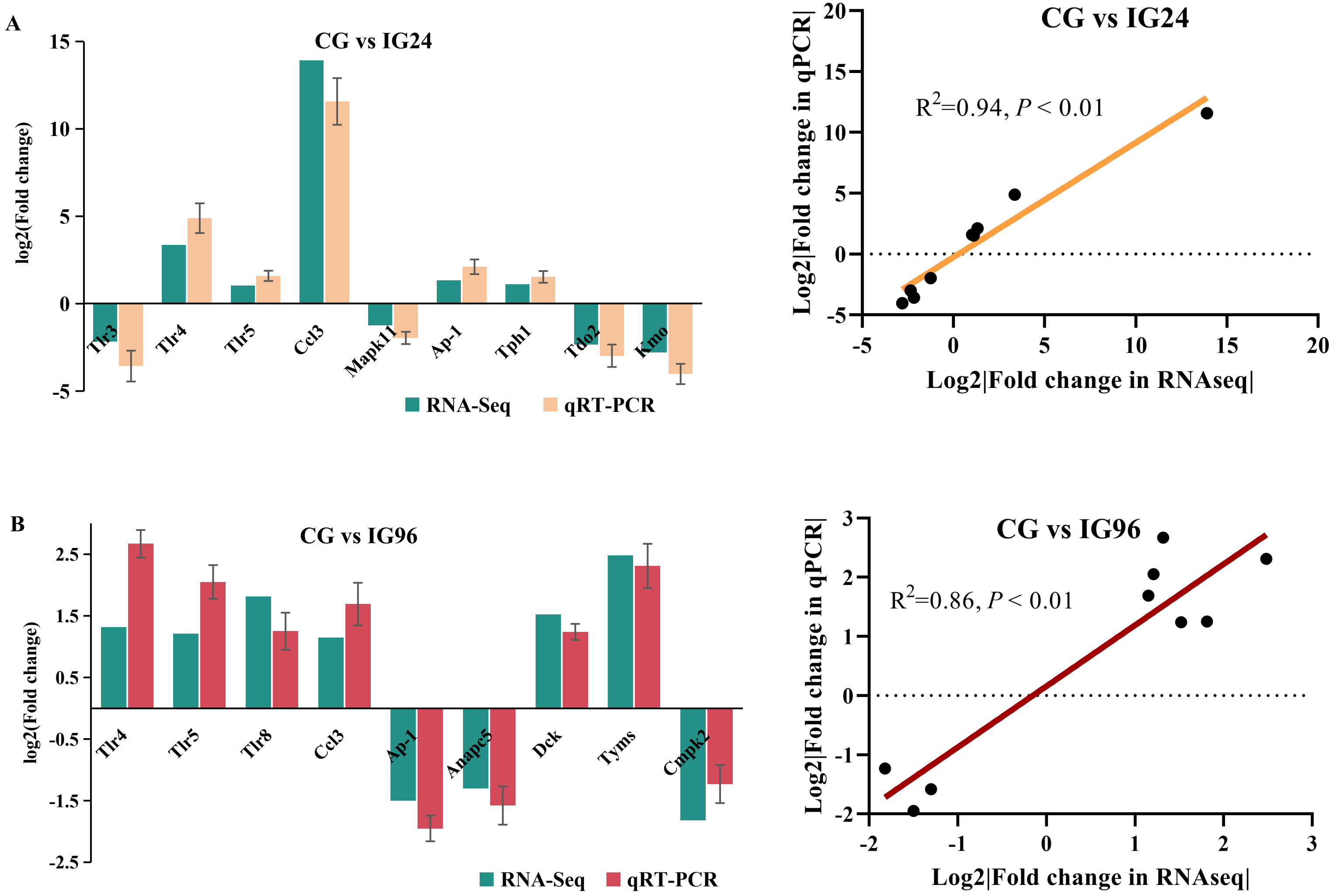

Supplement: Supplementary Figure 1 — Global analysis of differentially expressed genes (DEGs). (A) The correlation matrix of gene expression among five biological triplicates of different groups (n = 5). (B) PCA plot showing the variations of gene expression profiles in three groups. (C) Venn diagram analysis of DEGs in CG vs IG24, CG vs IG96, and IG24 vs IG96. (D) Number of DEGs (|log2(FoldChange)| > 1 and adjusted P-value < 0.05) in CG vs IG24, CG vs IG96, and IG24 vs IG96. Red and blue indicated up- and down-regulated genes, respectively. “CG” indicated the control group, “IG24”, and “IG96” respectively indicated the infected groups at 24 and 96 hours post A. hydrophila infection. [file DataSheet_1.pdf]
